# Survey of Visual and Force/Tactile Control of Robots for Physical Interaction in Spain

**DOI:** 10.3390/s91209689

**Published:** 2009-12-02

**Authors:** Gabriel J. Garcia, Juan A. Corrales, Jorge Pomares, Fernando Torres

**Affiliations:** Physics, Systems Engineering and Signal Theory Department, University of Alicante, PO Box 99, 03080, Alicante, Spain; E-Mails: jcorrales@ua.es (J.A.C.); jpomares@ua.es (J.P.); Fernando.Torres@ua.es (F.T.)

**Keywords:** visual servoing, force control, tactile sensors, multi-sensor control, robotics

## Abstract

Sensors provide robotic systems with the information required to perceive the changes that happen in unstructured environments and modify their actions accordingly. The robotic controllers which process and analyze this sensory information are usually based on three types of sensors (visual, force/torque and tactile) which identify the most widespread robotic control strategies: visual servoing control, force control and tactile control. This paper presents a detailed review on the sensor architectures, algorithmic techniques and applications which have been developed by Spanish researchers in order to implement these mono-sensor and multi-sensor controllers which combine several sensors.

## 1. Introduction

One of the main challenges in current research on robotics is the perception of the environment [1]. The first approaches developed to solve this problem were based on the definition of *a priori* models of the environment and the robot as precise as possible. This solution is only applicable when there are no changes in the environment and the precision of the model is sufficient for controlling the robot without significant errors. Nevertheless, these requirements are rarely fulfilled because robots usually perform their tasks in unstructured environments and their actuators are not perfect and accumulate errors when they perform their movements. Therefore, sensing becomes an essential component of current robotic systems, not only for exteroception (perception of stimuli from outside of the robot) but also for propioception (perception of the relative position of the parts of the robot). Sensors measure a physical property of the robot or of the objects in the environment and transform it into a signal which can be recognized and analyzed by the robot controller [2]. In fact, sensors are necessary to detect what is happening in the environment and how the robot is moving so that the robot's behaviour can be adapted accordingly. Thereby, robotic systems become more flexible and can be applied in different types of tasks and places.

Like humans, robots generally use the sensory information related to the senses of vision and touch in order to interact with the objects in their environment. On the one hand, vision provides the global information which is required to localize the objects in the environment and compute their relative spatial relations. This information can be used by the robot controller to avoid undesired obstacles or to reach the target objects which are necessary for the development of its tasks. On the other hand, touch provides the local information which is required to characterize the way the robot contacts the objects in the environment. This information can be used by the robot controller to manipulate the contacting object or to explore it so that its surface properties (e.g., shape, stiffness, heat…) are extracted for its identification.

Although the sense of vision is usually implemented in robotics through cameras, the sense of touch is implemented with force sensors and tactile sensors. For this reason, three different approaches are developed to control the robot's movements according to the information from these sensors: “visual servoing” (term defined by Hill and Park in 1979 [3]), force control and tactile control. Visual servoing combines techniques from image processing, computer vision and control theory in order to control the motion of the robot depending on the visual information extracted from the images captured by one or several cameras. Force control is based on the processing of the forces and torques which are transmitted between the robot and the objects when they come into contact. These force/torque values, which are registered by force/torque sensors installed in the robot, are usually used as inputs for control laws which guarantee that contact force/torque are regulated towards a predefined reference suitable for the development of the robotic task. Finally, tactile control provides a more fine-grained control of the interaction with the contacting object. In particular, tactile sensors detect different physical properties (pressure, deformation, stress, vibrations…) which describe more precisely the contact between the robot and the object. Therefore, tactile control is able to adjust the way the surfaces of the robot and the object come into contact so that the relative positions of these surfaces are optimal for the specific manipulation task.

These three control strategies determine the movements that have to be executed by the robot in order to perform the desired task according to the measurements registered by the corresponding sensors. This paper is focused on the description of the approaches proposed by Spanish researchers for the implementation of these sensory control strategies. The following sections of this paper present a deep review not only on the algorithmic basis of the implemented controllers but also on the sensors architecture and the real applications of the developed robotic systems. In fact, Sections 2, 3 and 4 describe visual servoing control, force control and tactile control, respectively. Nevertheless, as in human perception, the combination of different sensors provides a richer knowledge of the environment which is indispensable for developing more complex and precise tasks (e.g., assembly and disassembly tasks [4]). For this reason, Section 5 of this paper presents the different strategies which have been developed by Spanish researchers in order to put visual, force and/or tactile information together in multi-sensor controllers. Section 6 points out the conclusions of this review and discuss probable future advances on the sensory control of robotic systems. Finally, the last section is an appendix with a summary of all the research works which have been analyzed in this paper.

## 2. Visual Servoing Control

**Figure 1.**
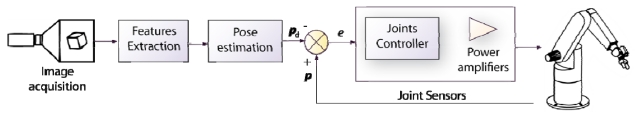
“Look and move” scheme.

Initially, robotic systems incorporating computer vision worked in open-loop (see Figure 1). This technique is known as “look and move”, *i.e.*, first the robot sees and recognizes the environment helped by a computer vision system, and after that, it performs the motion based on the data acquired in the previous step [5]. The vision system works in this approach as a pose estimator in order to obtain the required motion command to develop the task. Considering a task in which the robot must reach the position of an object in the workspace, the system does not check whether the object is reached; neither during the robot trajectory, nor once the robot reaches the final position. Thus, the “look-and-move” system supposes that the object position is not been altered from the moment the vision system obtains the object position through the vision computer system until this position is achieved by the robot.

An alternative to the previous approach is visual servoing [6,7]. Visual servoing is based on the use of visual information in the control loop feedback. More specifically, a visual servoing system uses the information acquired from a scene by one or more cameras connected to a computer vision system in order to control the robot end-effector pose with respect to a specific object in the scene. This closed-control loop approach permits to correct possible errors in the object position estimation obtained from the computer vision system. Moreover, it permits to change the robot trajectory in view of possible movements of the objects in the workspace.

**Figure 2.**
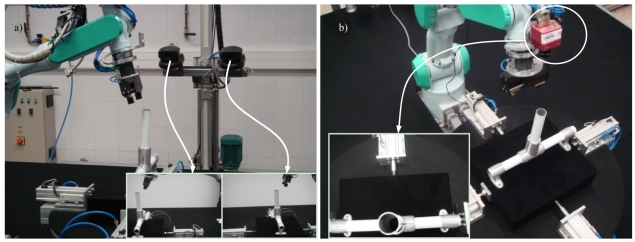
(a) Stereo eye-to-hand visual servoing configuration. (b) Eye-in-hand visual servoing configuration.

It is possible to place the camera in different positions. Independently of the kind of configuration employed, it is usually necessary to perform a camera calibration to determine its intrinsic parameters like the focal length, aspect ratio and image center. Generally, the camera is mounted on the robot end-effector, giving a more precise vision of the local environment of the task. Nevertheless, in order to perform other tasks, the simultaneous observation of the robot and its environment can be necessary. In this last case, the camera is usually mounted at a fixed position or over a second robot. In this case, the camera has no mechanical connection with the robot which is being visually controlled, but the relation between the camera and the robot base frame is known. Therefore, the cameras in a visual servoing system can be placed following two typical configurations: eye-in-hand (Figure 2a), or eye-to-hand (Figure 2b).

Sometimes the number of cameras may be greater than one to obtain a more confident geometric reconstruction of the environment. Figure 2a shows and example of a visual servoing system with eye-to-hand configuration that uses a stereo rig.

Another technique that is always developed for robot sensory control, independently of the sensor employed, are neural networks. Neural networks are generally implemented in two steps: training phase and classification phase [8]. Following this line, a feedforward neural network is used by Wells *et al.* in [9] to learn the complex implicit relationship between the pose displacements of the robot and the observed variations in global descriptors of the image, such as geometric moments and Fourier descriptors. The trained network may then be used to move the robot from arbitrary initial positions to a desired pose with respect to the observed scene. Reinforcement learning is a learning paradigm in neural networks [10] where the system receives a positive reward if the robot reaches the final desired position by observing the scene with the camera. If the system fails to take the robot to the goal pose, the system receives a negative reward. A smaller negative reward is sent to the system at every time step. Thus, the learner's aim is to obtain a mapping from the observed state to the actions to be performed in that state so that the accumulated reward is maximized in each state. El-Fakdi and Carreras describe a policy gradient based reinforcement learning technique applied to an autonomous submarine to perform cable tracking tasks [11]. Finally, Lopez-Garcia *et al.* [12] propose the use of Discrete time Cellular Neural Networks in order to get real time image processing. Discrete time Cellular Neural Networks are derived from cellular neural networks and feedback threshold networks. They are easily implemented on an integrated circuit by digital hardware technology, improving in this way the visual control speed.

The most widespread classification of visual servoing techniques depends on the input reference of the control loop. Sanderson and Weiss [13] established two different visual servoing approaches: position-based visual servoing and image-based visual servoing. From the visual features extracted by the computer vision system, the relative pose of the object to be reached with respect to the camera frame is estimated. This estimated pose of the object is compared with the desired one and the difference between both localizations is the controller input. On the contrary, in image-based visual servoing systems, the control is directly carried out in the image space. Thereby, the controller input is a comparison between the observed image features and the desired ones. The reference or desired features, **s**_d_, are expressed as features observed by the computer vision system in the desired location (points, lines, circles, corners…). The vision system is located in the feedback of the control loop, and deals with the extraction of this features during the task, **s**. The controller executes the necessary control actions in order to achieve the position where **s** = **s**_d_.

**Figure 3.**
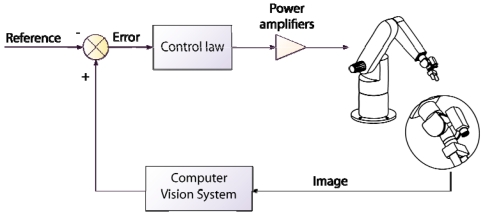
Direct visual servoing scheme.

Both techniques, position-based visual servoing and image-based visual servoing, are usually implemented in an external loop that provides an input to the robot internal controller. This approach corresponds with the indirect visual servoing. Contrary to indirect visual servoing, the robot internal controller is not present in direct visual servoing (see Figure 3). Thus, the internal joint controller of the robot is replaced by the visual controller, which uses the vision system data directly to control and stabilize the robot.

Before explaining the two main techniques of visual servoing, two papers describing a global distribute control system are presented. Cervera provides an object-oriented cross-platform network-ready environment for visual servoing simulations [14]. A cross platform language developed in Java, with native network capabilities, implements an agent-based architecture. Different agents construct a distributed model of visual servoing, where arm, control, vision and video transmitter can be located on different platforms. In [15] this agent-based architecture is used to obtain a visual servoing simulator for visual servoing learning. The developed simulator resolves the problems of previous visual servoing simulators, like the visual servoing toolbox for Matlab/Simulink [16]. Although a working version of the visual servoing toolbox is available, its development stopped due to the limitations of Simulink for object-oriented programming. Most of the functions needed to be coded in Matlab, thus its performance was too low for real-time simulations.

### 2.1. Position-Based Visual Servoing

**Figure 4.**
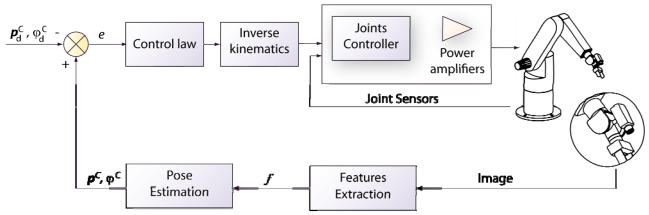
Position-based visual servoing scheme.

In a position-based visual servoing system (also called 3D visual servoing), the camera is a sensor used as a pose estimator. Both the current and the desired features are expressed in three dimensional (3D) coordinates (see Figure 4). The pose of the object with respect to the reference coordinate frame of the camera defines the current features. ***p***^C^ (position) and *φ*^C^ (orientation). The reference input is the pose of the object with regard to the camera frame at the robot desired position, ${\text{p}}_{\text{d}}^{\text{C}}$, $\varphi_{\text{d}}^{\text{C}}$. Finally, the difference between current and desired 3D poses defines the control error. Computing the mentioned poses from a set of measurements in one image requires knowing the camera intrinsic parameters and the 3D model of the object observed. This classical computer vision problem is called the 3D localization problem.

In this case, if the pose parameters are perfectly estimated, the robot trajectory is a pure straight line, while the trajectories of the image features are less satisfactory than in an image-based scheme as will be stated below. In the position-based scheme, it is impossible to ensure that the object will always remain in the camera field of view during the servoing task. This is the main problem detected in position-based visual servoing and thus, it is one of the most researched topics. Cervera *et al.* propose a system where the task is defined with respect to a different frame [17,18]. Typical position-based visual servoing systems define the pose of the object with respect to the camera coordinate frame. The frame proposed here is defined in such a way that its relative pose between the camera frame and the object frame at the final desired localization remains constant during the visual servoing task. Thus, trajectory of the object in the camera frame can be predicted, and it is likely to remain within the camera frame (in the absence of high calibration and/or model errors). The existence of a model of the object is the most constraining requirement of this approach.

The features usually used in a position-based control scheme are 3D points of the object expressed in the camera coordinate frame. In [19] Cervera *et al.* extend the features in order to avoid the previous knowledge of the object model. The use of a stereo configuration permits the authors to estimate not only the center of gravity of the blobs segmented in the scene but also the orientation of the principal axis of the blob. The behaviour is compared with the behaviour of an image-based visual servoing system, detecting a better behaviour in the image-based scheme than in the position-based not only in the image, but also in 3D. The position-based scheme proposed is sensitive to noise and calibration and a coupling between camera rotation and translation components has been found. This coupling can be improved by selecting different gains for these two components.

The modeling and implementation of a position-based system is presented in [20]. Here, the necessity of previous calibration is remarked. Bachiller *et al.* describe a modular scheme to implement different position-based visual servoing algorithms [21]. With an eye-in-hand configuration, the system has been tested with both, fixed and mobile objects. The problem of tracking a mobile object will be depicted in the Section 2.3 and represents an important topic research. The correct adjustment of the controller gains permits to perform the tracking of an object without the presence of a movement estimator. An optimal control approach has been used to perform the position-based task. The controller is designed so that the possible effects of actuator saturations are taken into account. A teleoperation application for the test of visual servoing schemes is presented by Wirz and Marin in [22]. The software is tested with a position-based visual servoing. The measured latencies confirm that Internet is not adequate to perform the visual features extraction on the client side. The estimation based on the acquired image requires sending this image through the net. The solution presented is to perform the visual features extraction on the server side. In this case, the transmitted information is smaller than sending the whole image.

Pose estimation of the object is perhaps the most important topic in position-based visual servoing research. It is important because the control input reference is defined through this pose estimation. Abderrahim *et al.* deals with this problem in [23]. Two different techniques are described for pose estimation: projective geometry approach and genetic algorithms: EvoPOSE. These techniques are tested in an application of satellite repairing in space orbit. A chaser uses position-based visual servoing to maintain the damaged satellite in the center of the image. The model of different satellites is required to perform the object identification and matching. The estimation of the pose of the object becomes an important issue in the case of direct visual servoing. As described above, in direct visual servoing the internal actuators' controller is replaced by the visual controller. The dynamic of the robot is necessary to obtain a robust control law. In [24], Vargas and Rubio describe a hybrid feature/position-based visual servoing where not only the geometric model of the object, but also the dynamic model of the robot are taken into account. Angel *et al.* describe an architecture designed to perform direct visual servoing tasks [25]. Specifically, this paper presents RoboTenis, a parallel robot designed to play table tennis. In this paper the authors present the design of the robot using ADAMS. The maximum velocities required to the playing tennis task are also analyzed. This work is expanded by Sebastian *et al.* in [26], where the position-based visual servoing system developed for the RoboTenis is described. Previous study of the physical requirements permits to achieve movements of 1 m/s. The high control loop velocities achieved with direct visual servoing make it adequate for performing this task. The proposed method takes into account the system delays and the saturation components. An estimation of the ball speed is necessary to feed back the control loop. A trajectory planner for the internal loop of the controller is described in [27]. The trajectory planner makes the system reach the target without exceeding the maximum acceleration and speed.

Montijano and Sagues describe a position-based navigation technique for a mobile robot [28]. They employ the images acquired from the camera mounted on the robot to obtain 3D information of the target position. The system simultaneously obtains metric localization and scene reconstruction using multiple homographies.

### 2.2. Image-Based Visual Servoing

Contrary to position-based visual servoing, image-based visual servoing is more suitable when a geometric model of the task to be developed is not available. In order to determine the control law in this approach, image features of the object in the visual sensor are used without the necessity of calculating their 3D localization, resulting in a more robust approach regarding calibration errors than position-based visual servoing.

**Figure 5.**
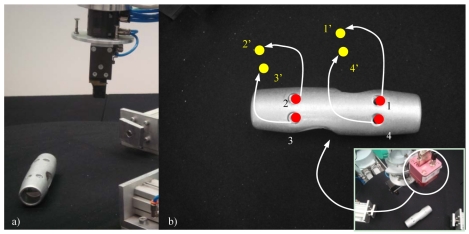
(a) Image acquired from an external camera. (b) Image acquired from the camera mounted on the robot end-effector.

Image-based visual servoing computes the control action in the two dimensional (2D) space of the image; and thus, this approach is also known as 2D visual servoing. The 2D visual features used in an image-based visual servoing system are usually basic geometric shapes (e.g., points or corners) which help to recognize without ambiguity the projection of a specific scene object. Figure 5 shows typical images acquired by an image-based visual servoing system.

**Figure 6.**
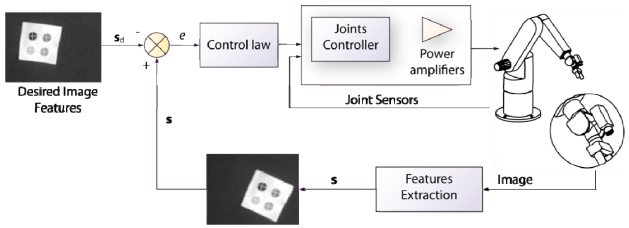
Image-based visual servoing scheme.

The image shown in Figure 5a is acquired by a fixed eye-to-hand configuration. The image shows a robot grip which is approaching to a metallic piece in order to grasp it. Both, the object and the robot are in the image space. Figure 5b shows an image obtained by an eye-in-hand configuration. In this image, the current (1, 2, 3, 4) and the desired (1′, 2′, 3′, 4′) visual features are highlighted. The difference in the image position of each set of features is the error, **e**, which the control system has to reduce progressively. The general scheme of an image-based visual servoing system is shown in Figure 6. The control actions of the image-based visual servoing system move the robot at each iteration reducing the error so that the visual features observed are nearer to the desired ones. Once the error function is cancelled (*i.e.*, the current and the desired features are equal), the task concludes.

As mentioned above, the loop control of image-based visual servoing systems is fed back with the visual features obtained from the images, without the necessity of determining the 3D pose of the object to reach. This involves an advantage with respect to position-based visual servoing systems, it makes the system more robust against calibration errors, and it reduces processing times.

The desired visual features, **s**_d_, are usually obtained offline from a desired final position of the robot in the scene. Image-based visual servoing controllers minimize the error between the visual features at the current position and the visual features at the goal position: **e** = (**s** – **s**_d_). To minimize exponentially this error **e**, a proportional control law is usually employed:

(1) $$\dot{\text{e}}=-\lambda \text{e}$$

where λ > 0 is a proportional gain.

In a basic image-based visual servoing approach, the velocity of the camera **v**_c_ is the input for controlling the robot movements. To obtain the control law, the interaction matrix or image Jacobian, **L**_s_, must be firstly presented. The interaction matrix is a matrix that relates the variations of the visual features in the image with the variations of the poses of the camera in the 3D space, *i.e.*, its velocity [29]:

(2) $$\dot{\text{s}}={\text{L}}_{\text{s}}{\text{v}}_{\text{c}}$$

From Equation (1) and Equation (2), the velocity of the camera for minimizing exponentially the error in the image is obtained:

(3) $${\text{v}}_{\text{c}}=-\lambda {\hat{\text{L}}}_{\text{s}}^{+}\;\left(\text{s}-{\text{s}}_{\text{d}}\right)$$

where ${\hat{\text{L}}}_{\text{s}}^{+}$ is the pseudoinverse of an approximation of the interaction matrix. The desired visual features **s**_d_ are constant during all the visual servoing task. This camera velocity is then transformed to obtain the velocity in order to be applied to the end-effector of the robot. To do this, the constant relation between the camera and the end-effector is used in a camera-in-hand configuration. If the system uses a camera-to-hand configuration, the relation between the robot base frame and the camera frame is usually known, and the forward kinematics provides the relation between the robot base and the end-effector coordinate systems.

In general, interaction matrix is a function of:

- Intrinsic camera parameters.
- The current value of the visual features in pixels.
- 3D information relating to the 3D points corresponding to the visual features (e.g., the distance from the camera to the corresponding object feature).

Determining the way in which the interaction matrix is updated in the control loop is not a trivial question due to the dependencies described above and represents the major topic of research in image-based visual servoing topic.

The simplest way to compute the interaction matrix is an offline update. In this case, interaction matrix is computed only once before the visual servoing task is started. The value of the visual features from which the interaction matrix depends is considered equal to the desired features. Therefore, the distance between camera and object is the distance in the final position. This approach reduces the quantity of computing operations during the visual servoing development and maintains the convergence [30].

Nevertheless, problems of convergence in large movements have been stated when using the desired visual features to compute the interaction matrix during the visual servoing task [31]. Furthermore, the evolution of the features in the image using this offline interaction matrix does not follow a straight line. Thus, the visual features may get out of the camera view, causing the failure of the task. Different approaches have been proposed to obtain a better behaviour than interaction matrix offline computation. Pari *et al.* present an online interaction matrix estimator based on the previous movements of the camera [32,33]. These papers include the epipolar constraint or fundamental matrix in the interaction matrix calculation. A comparison between this online estimated interaction matrix and the analytic interaction matrix is described in [34]. Echegoyen *et al.* [35] develop the equations to obtain the interaction matrix for an Aibo ERS-7 robot. They show the common procedure to obtain the analytic interaction matrix.

As mentioned above, the values that must be estimated during the visual servoing task in order to obtain a permanently updated interaction matrix are the intrinsic camera parameters and/or the camera-object depth. Usually, intrinsic camera parameters are computed offline in a calibration phase. This is valid for cases where the camera intrinsic parameters do not change during the visual servoing task. However, there are visual servoing tasks that require the high precision that zoom may provide. The use of the images acquired during a visual servoing task to perform the camera online calibration is developed in [36]. Pomares *et al.* describe a method based on virtual visual servoing which obtains the real intrinsic camera parameters from the images acquired up to the moment. The higher the number of images used to perform the calibration, the more precise camera parameters may be obtained. Nevertheless, a high number of images may compromise the visual servoing control time-step. The proposed solution is based on the definition of a calibration window, *i.e.*, the number of images sufficient to obtain a good calibration. In order to automatically determine the size of the calibration window an approach based on the Generalized Likelihood Ratio is proposed.

Next, some special topics related to image-based visual servoing research in Spain will be detailed: the visibility problem, the necessity for finding adequate visual features to perform the visual servoing task, the systems proposed to track predefined paths using image-based visual servoing, stereo visual servoing, and finally motion estimators to perform the correct traking of not static objects.

#### 2.2.1. The visibility problem

When the camera-object depth is online estimated, the evolution of the features in the image during the visual servoing task tends to follow a straight line [29]. This is not assured when the camera-object depth is offline estimated. Generally, offline estimation of the distance between the camera and the features is performed by choosing the distance in the final desired robot position. The offline depth estimation is less time consuming, and its implementation is easier than online estimation. Nevertheless, the visual features may get out of the image plane and thus, the visual servoing task may fail. The visibility problem has received particular attention in literature: a minimum number of image features must remain in the camera field of view during visual servoing. The most widespread solution to solve this problem is based on using potential fields to assure the visibility of all features during the control task [37]. Another solution to this issue is related to the intrinsic-free visual servoing approach [38] that zooms in order to keep all features in the field of view during the control task. Contrary to these solutions, Garcia-Aracil *et al.* proposed in [39,40] the concept of allowing temporary disappearance of image features during the vision-based control task. Thus, camera movements are not restricted as in the cases mentioned above or the zoom may be used to obtain a different view of the scene during the visual servoing task. To permit the appearing and disappearing of the visual features, they are weighted depending of their positions in the image space. The selection of the parameters of the weighted function is discussed in [41]

The use of panoramic cameras may avoid the appearance of outliers. Fisheye cameras provide panoramic vision whereby rich information is obtained. The work developed by Cervera *et al.* [42] deals with the detection of a human who enters the robot workspace. The motion equations of a 3D point are transformed to its spherical coordinates. Then, a simple relationship between the spherical coordinates and the fisheye image projection is used to compute the interaction matrix of an image point. The result is a more curved evolution of the features in the image and a better behaviour of the 3D end-effector path. The major advantage of the proposed visual servoing scheme is that the visual features do not leave the field of view thanks to the high angle of view of fisheye cameras. Its greatest setback is the necessity of a camera-object estimator (but it is a common problem in classical image-based visual servoing).

Online estimation of the camera-object depth is not an easy task. Moreover, it can introduce some undesirable behaviours. Cervera *et al.* introduce 3D information in the set of features of the control law [18,43]. They do not perform a 3D pose estimation of the visual features. Nevertheless, they compute the depth or distance from the camera to each characteristic point. With this new feature vector including 3D information, lineal displacement of the 3D object with respect to the camera is obtained. Thus, the object is most likely to remain in the field of view of the camera during the visual servoing task. A better behaviour is obtained using a 3D estimation of the object points from the intrinsic camera parameters and the estimated depth. Nevertheless, both proper camera calibration and 3D information obtained either by a pose estimation algorithm (assuming a CAD model of the object) or by a stereo visual system are required.

#### 2.2.2. The problem of finding adequate visual features

An important issue in the temporal efficiency of visual servoing systems is the complexity of the objects in the scene. Therefore, the objects are often converted to its simplest form to perform the image-based visual servoing. Techniques like marking the characteristic points on the object over a uniform background are usually used for the visual features extraction. The image processing is then merely a segmentation of these features and the computation of their center of gravity. The works developed by Pages *et al.* introduce external features to any object in the scene. They link to the camera a structured light emitter designed to produce a suitable set of visual features. The system is validated in a plane-to-plane task where only three degrees of freedom are considered. Depending of the visual features vector chosen, they demonstrate the decoupling of the interaction matrix only in local conditions (*i.e.*, positions near the desired location) in [44], or the global convergence in [45]. This last paper details the convergence study of the proposed visual servoing scheme. The global convergence of the system is ensured in the absence of calibration errors. Furthermore, the robustness, with respect to misalignment between the camera and the lasers, has been improved by defining an image transformation. Nevertheless, the system is only applicable to plane objects and only 3 degrees of freedom. The analytic stability of the previous mentioned system is achieved by using a new set of visual features described in [46]. Visual servoing does not bring any solution for positioning with respect to non-textured objects or objects for which extracting visual features is too complex or too time consuming. Pages *et al.* insist on a solution to the problem of visual servoing tasks with non-featured objects [47]. The problem is solved by projecting coded structured light to the scene. With the use of coded patterns, visual features are available independently of the object appearance. The classical image-based visual servoing control law is then applied using the points drawn on the object surface by the coded structured light. Experiments show that good results are obtained when the robot is positioned with respect to planar objects. On the other hand, the results when using non-planar objects show that the camera motion is noisier, slower and less monotonic. Camera and projector are not calibrated and thus, the online interaction matrix estimation is not possible.

Vargas and Malis describe a visual servoing system based on the homography decomposition [48]. The task of positioning the camera with respect to the plane cannot be achieved without any additional information due to the existence of two possible solutions in the homography decomposition problem. The analytic algorithm proposed here enables plane-to-plane positioning tasks by moving the camera in order to reject one of the two possible solutions because of the visibility constraint. A switching control strategy is employed once the false solution is discarded in order to achieve a smooth control action. Calibration errors have not been considered because the camera placed at the end-effector has been calibrated in a previous phase. Switching control is also employed by Lopez-Nicolas *et al.* in [49-51]. The technique descibed here is a visual control based on epipoles for mobile robots. Taking the epipoles as visual features does not require complete camera calibration or any particular knowledge about the environment. The epipolar based control is designed to avoid degeneracy due to short baseline when the robot comes closer to the target. Additionally, an alternative solution is proposed to avoid this problem by switching between the fundamental matrix and the homography model similar to the one presented by Merino *et al.* in [52]. In [53] Becerra and Sagues present the design of a control law that solves the problem of passing through a singularity induced by the epipoles maintaining bounded inputs.

Another example of the difficulty of finding an adequated set of visual features to perform the visual servoing task is presented by Ortiz *et al.* in [54]. The tracking of an underwater cable by an autonomous vehicle is a difficult task due to the occlusions by the algae or the sand. Ortiz *et al.* describe a method based on the prediction using a Kalman filter to correct the orientation of the autonomous underwater vehicle during the cable tracking.

#### 2.2.3. Image path trackers

Image-based visual servoing systems present singularities and/or local minima problems in large displacements tasks [29]. To overcome these drawbacks maintaining the good properties of image-based visual servoing robustness with regard to modeling and camera calibration errors, the tracking of a sufficiently sampled path between the two distant poses can be performed. This is the case of the techniques described below. Pomares and Torres present in [55,56] the movement flow-based visual servoing. The task is codified using a vector field (the movement flow) as an alternative to the classical movement control. The movement flow is a vector field that converges towards the desired trajectory. In general, the movement flow has the following properties: its vectors at each point of the desired image trajectory are tangent to it and those outside the trajectory aim to decrease the tracking error. Consequently, movement flow is a vector field that indicates the direction in which the desired features to be used by an image-based visual servoing system must be located. As the desired features depend only on the current camera position and the movement flow, the tracking of the trajectory is time-independent. When a robot interacts with its environment, most of the methods for tracking image trajectories proposed up to now fail [37,38] because they are time-dependent. Thereby, if the robot collides with an obstacle, the trajectory will not be correctly tracked. During the obstruction, the controller continues sending the following time references. When the obstruction ends, the robot keeps the time restrictions without tracking the trajectory correctly. Garcia *et al.* [57,58] describe an image path tracker with the same time-independent behaviour as the movement flow-based visual servoing described above or the path tracking system presented in [59], where the visual servoing system proposed to track the trajectory in the image deals with the tracking task as a sequence of positioning tasks. It uses the classical image-based visual servoing control law (see Equation (3)) to position the robot between two consecutive positions into the path. When the error is cancelled (the robot velocity is 0), the system guides the robot to the next position in the path. This process is repeated until the robot has visited all the references in the path. Thus, this system is not able to maintain a constant velocity during the tracking. Movement flow-based visual servoing presents some problems when high velocities are required. Moreover, it is not possible to specify the desired velocity at which the robot tracks the trajectory. The time-independent visual servoing proposed in [57,58] overcomes this limitation and the tracking velocity can be adjusted to a desired value. This last image path tracker uses virtual visual servoing to search the camera configuration that guarantees the desired velocity between two consecutive references.

### 2.3. Stereo Visual Servoing

Stereo rig configurations have been widely applied in the literature to obtain 3D information from the scene. The application of stereo vision in visual servoing was pioneered by Maru *et al.* [60]. Stereo visual servoing presents some particularities with regard to the monocular visual servoing. There are two cameras and thus, there are two images at each control loop iteration. From these two images, two different centers of gravity of a segmented blob corresponding to the same 3D point can be obtained. Cervera *et al.* describe in [61] and [62] different control schemes for a stereo visual servoing. By using 2D information they develop the interaction matrix for two cases. The first one computes the feature vector by expressing the visual features in the same coordinate frame. For this, the visual features are previously translated to a reference frame. The second 2D approach uses directly the measured position of the visual features in the two images. The interaction matrix is composed by stacking the interaction matrices obtained from each image as if they come from the same image. These two control actions are then compared with the techniques described in [43] which uses 3D information. The systems proposed are tested and they seem to be robust with respect to calibration errors. It has been shown that problems can appear if the relationships between frames are not properly taken into account. Particularly, it concerns some uncontrolled motions which can take the robot beyond its joints limits. Following the works described above, the same authors present a new feature vector, composed of the visual features measured in pixels and the disparity of the two images of the visual stereo rig [63]. Cervera *et al.* conclude that the use of 3D features allows the linearization of the interaction matrix and so a better joint decoupling. In [19] visual features are obtained from segmented image of objects. From each blob, its center of gravity and the orientation of its major axis of inertia are computed. The stereo rig is used for estimating 3D positions and orientations. This approach suffers from coupling between orientation and translation. However, this coupling is smaller than the observed in [63]. The authors conclude that a better study of the influence of noise and the camera calibration is necessary to improve the robustness of the stereo visual servoing schemes proposed.

Another stereo visual servoing system is presented by Recatala *et al.* in [64]. The proposed system controls only 4 degrees of freedom. Grasp points are used as control features in the design of the control law. The process of searching and tracking of the grasping points is also described, as well as the desired grasping pose, which is computed online. Sebastian, Pari *et al.* take into account the epipolar geometry of two fixed cameras overlooking the scene for online estimation of the image Jacobian [32-34]. The stereo rig is used for obtaining the epipolar constraints or the fundamental matrix computation. Mejias *et al.* propose the use of a stereo rig mounted on a helicopter to recover the height of the flight [65,66]. The visual features chosen to perform the visual control of the helicopter are the corners extracted using a Harris Corner detector or a SIFT detector. The flight tests performed deals not only with static but also with moving objects as posters over a car [67].

### 2.4. Tracking of Objects: Movement Estimators

Previous research on motion object tracking in real time like [68,69] has brought out the development of new algorithms designed to the processing of high velocity image sequences. The development of these algorithms, together with the recent improvement in the processing time of current computers, permits to obtain a better behaviour of the visual servoing systems. Generally, tracking techniques consisting of the use of marks or previous knowledge of the tracked objects are used [70]. Nevertheless, an agreement between processing velocity and flexibility has to be reached, making possible to perform a tracking in unstructured environments or even in situations where any of the components of the visual servoing system fail.

Bachiller *et al.* study in [21] several types of estimators, such as first and second order linear regression predictors, Kalman filter based on the assumptions of constant acceleration target motion and an auto-regressive discrete time model. These estimators have been used successfully for position estimation. However, they produce considerable errors when evaluating tracking performance for sinusoidal or triangular profiles of the target motion. Kalman filter is one of the most extended motion estimators. Pomares *et al.* [71] describe a tracking system based on the Kalman filter, which automatically adapts the amount of information to be processed, depending on the goodness of the tracking. Thereby, if the filter tracks the object suitably, the amount of information about each image to be processed by the filter will be reduced. On the contrary, if imperfections are detected, an adjustment is made in the tracking, thus increasing the processing time. Methods for the control of the trajectories of the moving objects are developed in order to avoid external factors and occlusions. Thus, in case of an occlusion, the position of the object is inferred statistically. In [72] the authors apply the motion estimator to a peg-in-hole motion task. Based also in the Kalman filter, it can also be found the work presented in [73] by Perez *et al.* The proposed approach (named Fuzzy Kalman Filter) is based on several cases of the Kalman filter and makes use of a Fuzzy predictor. The Fuzzy Kalman Filter decreases the tracking error in comparison with the classical Kalman filter for abrupt changes of direction and can be used for an unknown object's dynamics.

As it is described in [74], the estimation of the motion speed of a mobile object tracked by an eye-in-hand system can be obtained through the measures of the camera velocity and an error function. The information obtained from the images acquired in two consecutive iterations of the visual servoing task is used by Pomares *et al.* in [75] to obtain the homography matrix. The decomposition of the homography matrix can be used to compute the translation and orientation performed by the camera in those two positions. Thus, this technique computes the camera velocity in order to improve the tracking of mobile objects.

Motion estimators are not only used to perform a tracking of a mobile object. One major control problem of visual servoing is to cope with the delay introduced by image acquisition and image processing. Ángel, Sebastian *et al.* [25-27] add a Kalman filter predictor to solve this problem in the visual control of RoboTenis: a parallel robot developed to perform high velocities tracking of a small ball. Perez-Vidal *et al.* [76] develop a trajectory control-based scheme that takes into account the mentioned delay by properly including an estimator that predicts several samples ahead of time in the scheme.

Table 1 summarizes the visual servoing approaches developed by Spanish researchers which have been described in Section 2.

## 3. Force Control

In the current tendency where robot manipulators are supposed to be more and more autonomous, control of the physical interaction between the robot and the environment is absolutely necessary. A simple motion control (like those described in Section 2) is not sufficient to obtain a successful execution of the manipulation task. Force/torque sensors provide the robot manipulator with the skills required to interact with the objects located in its workspace. A force sensor is defined as a transducer that converts an input mechanical force into an electrical output signal. It is a critical component for extending the capability of manipulation and assembly, especially with contact tasks that require mechanical operations involving interaction with the environment or objects. Most force sensors in robot manipulators have a common basis: a strain gauge. A strain gauge is a sensor whose resistance varies according to the applied force [77]. It converts force, pressure, tension, weight, *etc.*, into a change in electrical resistance which can then be measured. Normally, gauges are categorized by their construction into four groups: mechanical, optical, electrical, and acoustical. Among them, the most important and widely used gauge is the electrical-resistance type, where small changes in dimension result in equivalent changes in resistance. The metallic foil-type strain gauge consists of a grid of wire filaments (a resistor) of approximately 0.025 mm thickness, bonded directly to the strained surface by a thin layer of epoxy resin. When a load is applied to the surface, the resulting change in surface length is communicated to the resistor and the corresponding strain is measured in terms of the electrical resistance of the foil wire, which varies linearly according to strain. The foil diaphragm and the adhesive bonding agent must work together in transmitting the strain, while the adhesive must also serve as an electrical insulator between the foil grid and the surface. Almost all of commercial six axis force/torque sensors use metal foil strain gauges bonded to strain rings as the sensing element.

**Figure 7.**
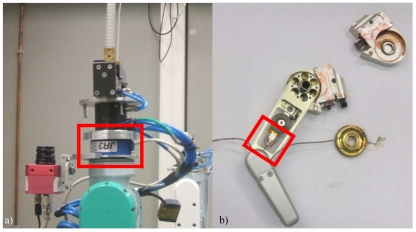
(a) Six axis force/torque sensor placed at the robot wrist. (b) Built-in strain gauge in a Barrett hand finger.

When a force/torque sensor is installed in a robot manipulator, it is frequently placed at the robot's end-effector [78] (see Figure 7a). This position of the sensor does not permit to directly measure the forces and torques exerted by the tooltip, as this tooltip is often placed over the robot wrist (between the sensor and the environment). If the weight of the tooltip cannot be negligible, it must be taken into account when reading the force and torques from the sensor. The same occurs with the inertia caused by the tool. This special issue related with the position of the sensor in the robot has motivated Garcia *et al.* to produce a set of papers with this topic research [79-81]. The idea developed in these works is to estimate the force/torque measures corresponding to the contact of the tooltip with an object. When the robot is performing a path, not only the interaction forces and moments at the contact point but also the inertial forces are measured by the wrist force sensor [82]. In order to compensate these inertial forces and moments, Garcia *et al.* propose the use of an inertial sensor also placed in the robot wrist. The combined action of these two sensors (force/torque and inertial sensors) is complemented with the joint sensors integrated in the robot. Based on the information obtained from these sensors, the authors describe a contact force observer. This observer not only obtains the exact forces and moments exerted by the tool over the contact surface, but also improves the performance as well as the stability and the robustness during the impact transition phase since it eliminates the perturbations introduced by the inertial forces. In [81,83] Garcia *et al.* extend this contact force estimator by adding a self-calibrating feature that allows an easy integration into any industrial setup. Fraile *et al.* [84] describe a force controller applied to a medical task. The task consists of bone drilling for long bone fracture repair procedures. This is a complex task that requires a high accuracy while avoiding excessive heating of the bone tissue. The force/torque sensor is located at the robot wrist and the surgical drill is placed below the force sensor. Due to the high accuracy required for this work, the weight of the drill cannot be negligible. To solve this problem, they compute the gravity term by using the forward kinematics of the robot.

Even though the great majority of research groups in Spain place the force/torque sensor on the robot wrist, there are some interesting works that present another configuration. A built-in configuration of the sensor is often chosen for walking robots [85,86] or flexible robots [87,88] (see Figure 7b). In [86,89], Montes *et al.* compute indirectly the forces between the feet and the ground using the measures obtained with the force transducers placed at candidate positions. These positions are selected at the design state of the robot's mechanical configuration with finite-element analysis. The best candidate positions are then tested, sensors are calibrated, and the results are experimentally evaluated. Flexible robots have a nice attribute in interaction tasks: the robot is able to collide with obstacles or humans in a soft way because of the flexibility of the links. However, the control problem of the flexible manipulators is not easy due to the vibrations appearing when the robot is performing a movement (e.g., [90]). Following this trend, Garcia *et al.* show a method to detect a collision by placing the force sensor at the root of the links [87]. The same approach uses Payo *et al.* in [88], placing the sensor at the beginning of the last link of the robot. The authors present a force controller for both free and constrained motions and design a collision detection algorithm only based on the measurement of the coupling torque at the root of the arm.

When a robot manipulator has to interact with its environment, motion control is not sufficient to assure a correct task execution [91]. There are two general techniques commonly used to control the interaction: passive interaction control and active interaction control [92]. The kind of interaction task defines the possibility of adopting one technique or the other. The former strategy implies that the robot is built in such a way that it can be compliant with the contact surface in an interaction task. Flexible robots are adequate to this kind of interaction control, as its links can be deformed to permit the adaptation without breakages. Frequently, the passive interaction control is performed by equipping the robot with a special-purpose compliant end-effector [93]. This compliant tool must be designed especially for a particular task. Passive interaction control does not require any sensor measuring the forces or the moments exerted by the robot. However, it is not adequate to performing tasks with large errors between the planned path and the compliant final path, as it is limited by the compliance of the robot. Furthermore, the lack of a force sensor prevents the passive interaction control from guaranteeing that high contact forces will never occur. Active interaction control schemes require the use of a force/torque sensor to measure and feed back forces and torques from the manipulated object.

According to the scheme of the control, active interaction control can be divided into two groups: indirect force control and direct force control [92]. The former achieves the control of the interaction with the motion control, without explicit closure of a force feedback loop; the latter permits to control the contact force and moments to a desired value due to the closure of a force feedback loop. Both of them will be detailed in the next subsections. The force control section is completed with a description of other control schemes using force/torque sensors in robot manipulation which cannot be grouped in the principal schemes.

### 3.1. Indirect Force Control

**Figure 8.**
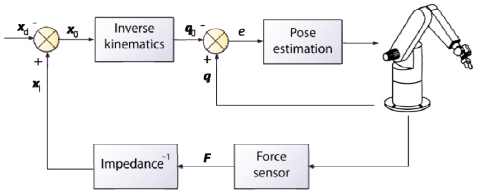
Impedance control.

One of the most used indirect force controllers is the impedance control [94]. The general scheme of this approach is shown in Figure 8. This control is designed to regulate the mechanical impedance of the robot, *i.e.*, the dynamic relationship between the exerted force and the movement error. Admittance control differs from impedance control in the way that the robot reacts to the motion deviation. Impedance control generates forces whereas admittance control generates a deviation from the desired motion. In general, impedance control provides a better behaviour with softer movements [95]. However, a dynamic model of the robot is required in order to guarantee the stability. Thus, both the robot and the environment are modeled as an equivalent mass-spring-damper system with adjustable parameters in the case of impedance control.

Admittance control is also called position based force/impedance control in the literature, indicating that the desired behaviour is implemented by means of outer loops around an inner position or velocity loop. It is the strategy chosen by Galvez *et al.* in [96]. In this paper, the authors present a design of a legged-robot with a built-in force/torque sensor configuration. Their approach differs from most admittance control algorithms in that they place the force controller in joint space. Force distributions in the legs as well as the contact surface orientation are computed online to obtain the input force component of the controller. The control presented reduces the risk of foot slippage.

The works presented by Garcia *et al.* [79-81,83] deal with the problem of filtering the force/torque signal from the sensor in order to obtain the exact contact forces and moments exerted by the robot. They do not describe a controller, but an estimator of the contact force and torque exerted by the manipulator to the environment. Thus, to validate the contact force estimator, they implement an impedance control. The model used to design the impedance controller, which includes the robot dynamic and the tool, is considered for the three Cartesian task-space axes.

One important issue in the research of a certain control topic is to have an open architecture which provides the basis for the easy and fast development of different control schemes. Garcia *et al.* present in [97] an open software architecture for an industrial manipulator that permits the easy implementation of model-based and sensor-based control concepts. It allows the integration of standard industrial components, and has been tested for a robotic interaction task with two different control schemes: impedance control and hybrid control. Another test platform is designed and developed by Valera *et al.* in [98]. The final aim of the test platform is to analyze the mechanical behaviour of car seats during passenger ingress and egress. A dummy held by a robot is intended to reproduce the forces applied by a human who is sitting down and standing up from the seat. The test platform provides two different control architectures: one based on proprietary software (which implies having less sampling velocity) and another based on open software (which is able to operate at low-level and thus, provides better sampling velocities). The force control schemes that have been tested in the test platform are the impedance control and different schemes of direct force control.

### 3.2. Direct Force Control

Direct force control is intended to control the forces and moments in a robot interaction task. There are several strategies to perform this control of the forces. A pure force control is used in tasks where only the forces have to be regulated. The motion is directly controlled by the force input and the final position of the end-effector of the robot is not important for the correct completion of the task. ROBOCLIMBER is a legged-robot able to climb and walk. It implements a simple control scheme where the prismatic joints of the legs are controlled directly by the measured ground-reaction forces with a proportional force controller [86,89]. The same direct pure force control is used by Puente and Torres in [99]. The measured force is used by the control to adjust the velocity of the end-effector of the robot in a screwing task. A Kalman filter removes the force peaks due to the noise in the reading of the force/torque sensor. A threshold force indicates the end of the task. In these tasks, the only command for the robot is the desired force, and the responsibility of the control is to move the tool in order to achieve this desired force. It is a first approximation to direct force control. However, when both motion and force must be controlled pure force control is not sufficient.

Hybrid force/motion control is the most extended direct force control scheme. The general scheme of hybrid force/motion control was firstly proposed by Raibert and Craig [100]. It basically consists in selecting the direction on the workspace that have to be controlled by the motion and the directions where the interaction force exerted by the robot has to be controlled. Thus, the system presents two separated control loops for motion and force, respectively. Therefore, in those directions assigned to the motion control the current position/velocity is determined, whereas in the directions assigned to the force control the interaction forces between the robot and the environment are measured. One very clear example can be found in [84] where Fraile *et al.* propose a hybrid force/motion controller to adjust the velocity and the force of a drilling task in a bone. The force control is only required for the drilling axis, a binary diagonal selection matrix **S** is adjusted to limit the force control only to the Z axis of the drilling tool. The rest of the directions are controlled by the motion control. Hybrid force/motion control schemes control the constrained task directions with the force measured by the sensor whereas the unconstrained task directions are controlled by the motion controller. Based on the task frame formalism (first devised by Mason [101], and then reviewed in [102]), it is developed the force controller described by Prats *et al.* [103]. Task frame is a Cartesian coordinate system attached to the object the robot is going to interact with. This frame defines the task according to the natural constraints imposed by the environment. Again, a hybrid force/motion control is used to control both the interaction forces and the motion. Forces measured in the task frame are then controlled by an impedance controller in the direction perpendicular to the contact surface. The velocity, given in the task frame coordinates, is finally composed by the independent subspaces components of the motion controller and the impedance controller velocities. Therefore, the robot executes the task by trusting its estimation of the task frame, but locally it modifies the trajectory by an impedance approach, in order to avoid excessive forces. Amat *et al.* [104] propose a hybrid position/force controller in human robot interaction tasks. The system uncouples the movements based on the obtained forces and moments during the interaction. The system is tested in service tasks like shaving and feeding. Once the directions have been uncoupled, the systems apply a force controller to the directions perpendicular to the face and a motion controller to the rest.

Switch control is often applied to tasks where there is an unconstrained motion of the robot arm followed by a constrained motion [105]. Different controllers are implemented for free motion or contact motion. Hence, a contact force estimator is required in order to precisely separate both states. This is the technique chosen by Payo *et al.* in [88] for controlling free and constrained motion of a single link flexible robot. The flexible robot is modeled as a lumped-mass where the arm is assumed to be very lightweight and the totality of its mass is concentrated at the tip. A collision estimator switches from a trajectory for free motion to a trajectory for constrained motion when a collision is detected. The control law is described as a modified PID controller.

### 3.3. Other Control Schemes Using Force/Torque Sensors in Robot Manipulation

Although the most extended control schemes belong to one of the two groups described before, there are other techniques that offer excellent results in a specific task. Fine motion planning is a commonly studied issue in the literature. The most commonly used example to define a fine motion task is the insertion of a peg in a hole. It is a precise task that cannot be planned previously, as uncertainties can avoid the complete execution of the task. The position of the end bar is computed by forward kinematics of the robot and by the matrix which relates the end bar frame with the robot end-effector frame. Errors in the matrix relating both frames or in the calibration of the robot may lead to a failed task completion. Contact between parts has to be monitored, and different actions are needed to perform a correct insertion. Suarez *et al.* [91] build a plan by considering all the possible contact states in simple planar tasks. They do not make use of the magnitude of the measured force. The only measure used is the direction of the reaction force. Depending of the direction of the reaction force, the effect of friction and the geometric uncertainty, a set of different states is defined offline. The system determines during the task which state represents the current situation. The main problem of the technique described in this paper is that it is based on geometric models, which become complex for non-trivial cases especially in three dimensions [106]. The states stored in an offline stage in the previous paper [91] can be used by a neural network in a perception-based learning approach [78]. A self-organizing map is used to learn the different contact states in an unsupervised way. The learned states allow the system to classify the current state and perform the corresponding motion. The method is proved to work properly in complex interaction tasks where geometrical analytical models fail. A reinforcement learning algorithm improves the learning phase of the neural network. Cervera and Del Pobil extend the work presented in [78] using a Q-learning algorithm which maximize the reinforcement signal [107]. A feature extraction neural network complements the learning algorithm. The implemented approach exhibits good generalization capabilities for different shapes and locations of the assembled parts.

Table 2 summarizes the force control techniques developed by Spanish researchers which have been described in Section 3.

## 4. Tactile Control

Tactile sensing is a technique which determines the physical properties of objects through their contact with the world [108]. Tactile sensors are one of the most varied families of sensors because they are based on different physical properties, they are implemented by different sensor technologies and they are installed in the robot according to different configurations. Table 3 shows a summary of these different features of tactile sensors.

Tactile sensors can be classified into two main groups according to the measured property: static and dynamic tactile sensors. Static tactile sensors analyze the physical properties of two static contacting surfaces while dynamic tactile sensors are based on the study of the variation of the physical properties of two contacting surfaces which are moving. On the one hand, static tactile sensors are generally based on the measurement of the normal pressure over the contacting surfaces or their deformation [109]. On the other hand, dynamic tactile sensors measure the vibrations or changes in stress of the contacting surfaces [110] in order to determine when slippage between the contacting surfaces takes place.

**Figure 9.**
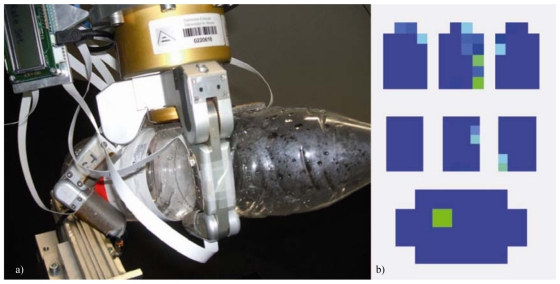
(a) Pressure sensing arrays installed on the fingers and the palm of a Barrett hand; (b) distribution of pressure values registered by the tactile arrays.

Tactile sensing is developed by arrays of sensors which emulate the human skin and obtain a distribution of the measured property over the contacting surface. Each of these sensors is implemented through different technologies [111,112]. Pressure sensing arrays (see Figure 9) are generally implemented through capacitive sensors, piezoresistive sensors or optical sensors. Skin deflection sensors are generally implemented through conductive rubbers, arrays of piezoresistive strain gauges or optical sensors. Finally, dynamic tactile sensors are implemented through piezoelectric transducers and accelerometers which are very sensitive to vibrations or piezoelectric stress rate sensors.

Tactile sensors can be installed in the robot according to two main configurations: intrinsic and extrinsic [113]. Intrinsic tactile sensors are integrated inside the structure of the robot while extrinsic tactile sensors are installed over the surface of the robot. In particular, intrinsic tactile sensing is applied on robotic hands and is generally based on a fingertip force/torque sensor implemented with strain gauges which measures the forces and torques that are applied over the last phalanx of each finger. If the geometry of the fingertip is known, the force and torque values obtained by intrinsic sensors can be used to compute the position of the contact points [114]. The implementation of extrinsic tactile sensors is significantly more varied (pressure sensing arrays, skin deflection sensors and dynamic sensors) and relies on the direct measurement of physical properties (pressure, force, deformation and vibration) over the contact surfaces. Nevertheless, the most common extrinsic configuration in robotics is based on the installation of pressure sensing arrays over the inner faces of the fingers of a robotic hand, as shown in Figure 9.

Despite these differences, all tactile sensors share a common property in robotics: they analyze the direct contact between the robot and the objects of the environment in order to adapt the robot's reaction to their physical properties. In particular, the robot can adapt its behaviour to the changes in the environment by processing the tactile information according to two different aims: object identification and manipulation control. On the one hand, the properties of the objects (texture, shape, compliance…) extracted from the robot's tactile sensors can be used to categorize the objects into different classes which have a specific reaction of the robot associated with them. Thereby, the robot's behaviour is adapted to the class of object which is touching at each moment. On the other hand, the measurements obtained from the tactile sensors can also be applied directly into the robot controller. In this case, a tactile-driven robotic control is developed and no identification of the contacting object is needed. The next sections present the research developed in Spain related with these strategies for controlling robots through tactile sensing.

### 4.1. Tactile Sensing for Object Identification

The identification of the object which the robot is touching can be implemented by two different techniques: geometric modeling and neural network classification. The first approach is based on the definition of a geometric model of contact which determines how the contacting surfaces of the robot and the object interact. The result of this interaction is registered and quantified by the tactile sensors which are installed on the robot. An estimation of the geometric model of the object's surface can be obtained from the combination of the known geometric model of the robot's surface with the interaction response measured by the tactile sensors. Galvez *et al.* [115] develop a intrinsic tactile sensing method which computes the normal vectors of the contacting surface and the shape of the object from a geometric model of the robot. This method is applied on an eight-legged pipe crawling robot in order to guarantee that there are no foot slippages, regardless of the pipe's shape. A five-axis force/torque sensor is installed at the last link of each leg and registers the forces and moments which are transmitted from the contact point with the pipe to the foot. First of all, the position of the contact point is computed from the measured force/torque and the geometric dimensions of the leg. Afterwards, the normal vector at the contact surface is found in order to model the local shape of the object's surface at the contact. Finally, a quadratic optimization strategy implements the friction cone inequality constraints corresponding to the computed normal vector in order to obtain the force distribution which guarantees the robot stability during the crawling process.

The other most commonly used technique for object identification from tactile data is neural network classification. In the training phase of the neural network, some known tactile data are applied to the network in order to infer the mapping between these data and the desired classes. The classes can have been established beforehand or can be generated automatically and they correspond to the most representative values for the physical property measured by the tactile sensors. After the training phase, the neural network is able to categorize new input tactile data into a specific class according to the previously inferred rule (*i.e.*, classification phase). The next two paragraphs present the two approaches which have been implemented by Spanish researchers to classify objects according to their tactile data (in particular, surface shape and stiffness) processed by neural networks.

Jimenez *et al.* [116] develop a method for classifying the shape of the objects grasped by a two-fingered gripper with a 16×16 tactile array of conductive rubber. The implemented method is organized into three phases: noise cancellation, tactile image preprocessing and classification by a learning vector quantization (LVQ) neural network. The noise cancellation phase filters the tactile images (matrices of pressure measurements) in order to reduce their electronic noise and thus improve the results of the classification process. The image preprocessing phase computes the translation and orientation of the object in the tactile image and then transforms the image so that all the objects are placed at the center of the image with the same orientation. This transformation is necessary because neural networks are very sensitive to changes in position and orientation. The last phase of the method classifies the transformed tactile image into four different classes of local shapes (flat, edge, cylindrical and spherical surfaces) by applying a LVQ neural network. This information about the shape of the object is finally analyzed by the robot controller to choose the most suitable manipulation strategy for the object.

Pedreno-Molina *et al.* [117,118] present a neural estimator of the stiffness of an object which should be grasped by a robot with a predefined force threshold. It is composed of three neural models: a displacement neural estimator, a motor command generator and a robotic joint controller. The first model is based on a neural network organized as a two-dimensional topographic map which relates increments in joint position displacement to the force applied on the contact. During the training phase, several objects with different stiffness come into contact with the robot in order to fill this two dimensional map with pairs of joint displacements and forces. Thereby, when a new object contacts the robot, the neural network interpolates the joint displacement required to apply the desired contact force from the learned curves whose coordinates where stored in the topographic map. The second neural model is based on a VAM (Vector Associate Map) which relates the joint position obtained by the first model to the corresponding muscles tensions which are required to control the robotic finger joint. The third model receives as inputs the previous tensions and generates the final commands for positioning the robotic finger. In this way, the neural architecture adapts the joint angles of the robot to the stiffness of the object in order to apply the desired contact force.

### 4.2. Tactile Sensing for Manipulation Control

In the approaches described in the previous section, tactile information is processed in order to classify the objects according to a physical property (shape, stiffness…). After this classification process, the robot controller chooses the most suitable reaction for the class of the object. Therefore, the robot controller performs an identification of the object depending on its tactile properties and reacts accordingly. Nevertheless, tactile information can also be used as input for the control law of the robot. In this case, there is neither object identification nor tactile information interpretation, but a mathematical processing of tactile data.

Morales *et al.* [119] implement a force-pressure control law which is applied on a robotic manipulator (UJI Service Robot) in order to grasp a book from a bookshelf. The manipulator has a force/torque sensor installed at its wrist and a Barrett hand with three fingertip pressure sensing arrays at its end-effector. The control law has two loops: a force loop and a pressure loop. The force loop verifies that the force applied on the book and registered by the force/torque sensor is enough to extract the book from the bookshelf. The pressure loop is dependent on the force loop and verifies that the pressure over the book's surface is not too high. When the applied force is not high enough, the force loop will increase the force until the pressure loop detects a high pressure value. Then, the fingertip is moved so that the contact surface between it and the book is increased. After the contact surface is increased, the applied force can be also increased without any risk of book or tactile sensor damage due to high pressure values. When the force applied by the fingers of the robot hand matches the desired value, the fingertip will move backwards in order to turn the book around its base without slippage risks. Thereby, the book can be extracted from the bookshelf.

Rodriguez-Cheu *et al.* [120,121] develop a control strategy which detects important grasping events for controlling the manipulation tasks performed by a prosthetic hand. The prosthesis is equipped with static and dynamic tactile sensors in the index finger and thumb. In particular, two FSRs (Force Sensing Resistors) cells are installed on the inner part of each finger, an accelerometer is installed on the outer part of the fingertips and a piezoelectric sensor is installed in the lateral part of each finger. The FSRs sensors measure the tactile pressure which is applying each finger on the grasped object, the accelerometers detect vibrations in the metallic structure of the finger and the piezoelectric sensors detect vibrations in the covered latex of the prosthesis. The measurements of the dynamic sensors (accelerometers and piezoelectric sensors) are combined by a binary function that detects sliding. This sliding detection function from the dynamic sensors, the tactile pressure measured by the FSRs and the grasping forces which are obtained as proportional values of the fingers actuators current are used as inputs to the control algorithm together with the EMG user signals. This control algorithm adapts the grasping operation ordered by the user through the EMG signals depending on the sensor signals (tactile pressure, grasping force and slipping detection). In addition, the control algorithm generates a suitable electro-stimulation signal which informs the user about the grasping process. This feedback allows the user to take part in the whole process of the grasping and improves the flexibility and controllability of the prosthetic hand.

Maldonado-Lopez *et al.* [122] have also developed an integrated tactile system which detects slipping in dexterous manipulation tasks. This system is composed by three main components: a 16 × 16 array of piezoresistive tactels, a signal conditioning circuit and a FPGA. The sensor array changes the resistance of their components according to the pressure applied to them. The signal conditioning circuit transforms the output of the sensors into a digital signal which can be processed by the FPGA. Finally, the FPGA implements an algorithm which detects slipping from the number of pulses of the digital signal within a specific time interval. If the FPGA detects slipping, it sends an alarm to the controller PC so that the manipulation task can be changed accordingly. The implementation of this slipping detection algorithm in a FPGA implies an improvement in the efficiency of the overall real-time manipulation system.

Table 4 summarizes the tactile control strategies developed by Spanish researchers which have been described in Section 4. In the next section about multi-sensor control, other tactile control strategies which are combined with vision and force are described in detail.

## 5. Multi-Sensor Control

**Figure 10.**
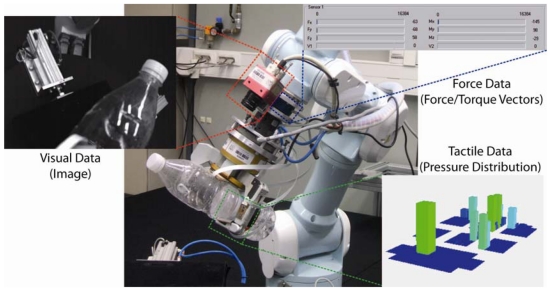
Example of a robotic task, consisting of grasping a bottle of water, which requires a multi-sensor control strategy.

Previous sections have presented the research work which has been developed by Spanish researchers in visual servoing, force control and tactile control separately. However, the combination of the global information registered by visual sensors with the local information registered by force and tactile sensors enables a more complete knowledge of the environment. In other words, visual information provides the robotic controller with an overview of the objects that compose the environment and force/tactile information provides the controller with a detailed description of the contact with them. Both types of information are required for complex tasks where the robot needs to manipulate objects in the environment. For instance, Figure 10 shows a manipulation task performed by a robotic manipulator with a Barrett hand which requires the combination of multiple sensory data: visual information (images) from an eye-in-hand camera, force information (force/torque vector) from a wrist force/torque sensor and tactile information (normal pressure distribution) from a tactile array. The following sections describe the main control techniques which have been implemented by Spanish researchers in order to solve this multi-sensor combination. In particular, Section 5.1 presents the multi-sensor control systems only based on visual and force control and Section 5.2 presents the multi-sensor control system which combine the three types of sensors.

### 5.1. Visual-Force Control

Visual/force control schemes are commonly derived from the position/force control schemes detailed in Section 3. The only difference is the presence of a computer vision system that usually performs the control of the position of the robot as the position control does. Therefore, the control schemes have to be modified to take the visual servoing component into account. The most employed motion/force control techniques like impedance control or hybrid control are also adapted to obtain a visual/force control. Moreover, a shared control scheme where both, force and image are fused in the image space is also studied by the Spanish researchers. Next, the different visual/force control schemes and their applications will be detailed.

#### 5.1.1. Visual/force impedance control

**Figure 11.**
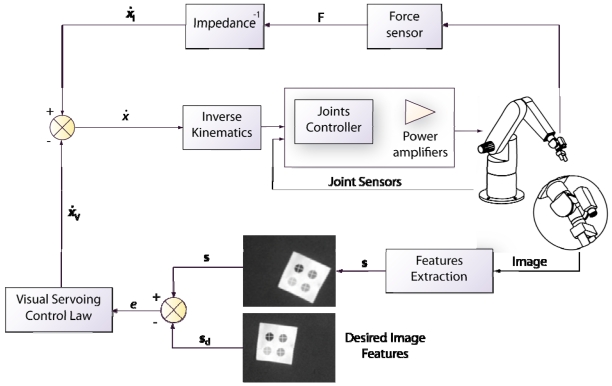
Visual/force impedance control.

The scheme of the visual/force impedance controlled is derived from the scheme of the general impedance control depicted in Figure 11. The measured force is used to obtain a velocity that modifies the velocity obtained by the visual servoing system. This velocity is then the input to the joints controller in an indirect control strategy.

Using impedance control to combine visual and force data for the interaction phase, and a general control that weights each of the sensory control, Pomares *et al.* describe in [72] a multi-sensor controller to perform a peg-in-hole motion task. The system is composed of three different components: a stereo rig in an eye-to-hand camera configuration, an eye-in-hand camera configuration, and a force controller. The control action is the weighted sum of the actions provide by each control subsystem. The weighting parameters depend on the state of the task. For example, in the reaching phase, the position-based visual servoing has a greater weight than the eye-in-hand image-based visual servoing system. Pomares *et al.* use a position-based impedance control system called accommodation control in which the desired impedance is limited to pure damping [123]. The main contribution of the proposed method consists in a modification of the image trajectory which assures the robustness of the method when errors in the camera intrinsic parameters appear. For this, homography decomposition is used to recover the camera motion between the current (constrained by the interaction forces) and the desired positions. From this camera motion it is possible to project in the image the new desired features for the visual servoing system. The same multisensorial control technique is employed in [124] to perform the interaction task. The paper presents a robust method to detect a change in the surface of the touched object. Camera motion is recovered from the homographies, and a method based on structured light detects the exact moment when the surface changes.

#### 5.1.2. Visual/force shared control with an external force loop

**Figure 12.**
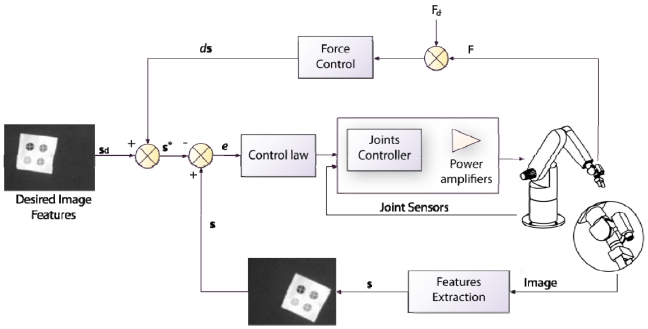
Shared control with an external force loop.

A shared control strategy permits to control all the directions of the task with the information of all the sensors of the system. Opposite to the scheme presented in the last subsection, here the force may be regulated. Therefore, this is a direct force control scheme. The force control modifies the desired features in the image, so that an internal visual servoing control loop is surrounded by an external force loop (see Figure 12).

Pomares *et al.* employ a shared control technique to fuse the information acquired from force and visual sensors [125]. The measured force permits to modify the image trajectory when contradictory actions appear. To perform this task, the normal contact plane and the online computed intrinsic parameters are employed. The camera velocity is the weighted sum of the two control action. These weighted values are updated online using the Generalized Likelihood Ratio, giving more influence to the force component when changes in the surface are detected. The visual-force controller is then applied to a disassembly task using an eye-in-hand camera configuration and a wrist-mounted six axis force/torque sensor. This same technique is employed by Prats *et al.* in [126,127]. The desired visual features for a visual servoing are modified by an external force control loop. When the end-effector is not in contact with the external environment, the output of the force controller is null and the robot is controlled according to the vision-based controller output. Since the force control only acts on the reference trajectory, conflicts between force and vision controllers are avoided. This controller is implemented for a robot arm placed on a mobile robot which is asked to open a door of a wardrobe. In order to achieve a position to start the manipulation, the robot is driven by a position-based visual servoing system. The vision/force coupling approach where the force control loop is closed around an internal vision control loop in a hierarchical way is employed in a manipulation task again by Garcia *et al.* in [58]. As in the papers described above, the force controller modifies the desired visual features. Nevertheless, in this paper the modification of the image features is achieved through the force-image interaction matrix [128]. This matrix relates the variations in the forces to the variations in the visual features. Thus, regulation of the forces in an interaction task may imply changes in the desired visual features. Moreover, the approach described in [58] uses a time-independent image path tracker based on virtual visual servoing to follow a predefined path. The autonomous change of a faulty bulb in a streetlamp is used to validate the proposal. The same technique is then applied by Gil *et al.* in [129] for a global metallic structure assembly task. The application of this shared control technique to the modification of the desired path in image path trackers is also applied in [130]. In this paper, Pomares and Torres employ a simple force controller which modifies the velocity value obtained by the movement flow-based visual servoing.

#### 5.1.3. Visual/force hybrid control

As described in previous sections, a motion/force hybrid control permits the control of complementary directions using different sensors. When the motion is managed by a visual servoing system, a visual/force hybrid controller is defined. This is the case of the controller described in [131]. The UJI librarian robot is a robot designed by Prats *et al.* to assist library users in a library. The robot has to search and extract the requested book. In [132] the algorithms used for the localization and manipulation of the book are detailed. The book manipulation is performed as stated above with a hybrid visual/force controller. Two degrees of freedom are considered. The perpendicular direction to the book is controlled with the force information, whereas the parallel direction is controlled with an image-based visual servoing by using the edges of the book as visual features.

### 5.2. Visual-Force-Tactile Control

The combination of visual, force and tactile information is the most complete strategy for controlling robots which interact with the environment. On the one hand, visual sensors obtain the global information necessary to detect and approach new objects in the environment. On the other hand, force and tactile sensors provide the local information required to grasp and manipulate the objects that have been previously detected by the visual sensors. These local and global data are generally combined in the literature by applying two different strategies: neural networks or hybrid control.

Pedreno-Molina *et al.* [133] present a visual-tactile-motor control system based on neural networks which guides the manipulation tasks performed by a robot arm with a parallel gripper. Firstly, a stereo-head guides the robot arm towards the target object and places the gripper over it. Next, the robot controller guides the manipulation process by applying a self-organized neural network with a VAM structure over the pressure images registered by the tactile arrays installed on the gripper. In particular, this controller aims to make coincident the centroid of the tactile image generated by the contacting object with the center of the tactile sensor surface. The training phase of the neural network is used to find the matrix relation between the movements of the centroids in the tactile images and the increments in the robot joints positions. Thereby, the neural network is able to compute the joints displacements which are required to position the centroid of the tactile image in the center of the tactile sensor. In [134] this control system is extended by adding a VAM neural network for the visual system. In the learning phase of this network, the relation between the visual projections into the cameras and the joint positions is computed. This relation is applied by the new visual-motor controller in order to reduce the distance between the image projection of the object and the robot gripper so that the robot is finally positioned over the object. When the object comes into contact with the gripper, the controller gives priority to the tactile information and applies the tactile VAM neural network described above in order to perform the manipulation task. Thereby, visual information is used to reach the object while tactile information is used to manipulate it.

Prats *et al.* [135] present a position-vision-tactile hybrid control which is modified by an impedance force control in order to open a sliding door with the UJI service robot. As stated before, this robotic platform is composed by a robotic manipulator with a three-fingered Barrett hand, a wrist force/torque sensor, a pressure sensing array for each fingertip of the hand and a camera in an eye-to-hand configuration. Firstly, three independent controllers are executed: a position controller, a vision controller and a tactile controller. The position controller obtains the initial estimations of the robot pose and the target object pose from sonar/laser measurements and applies them on a proportional position-based control law in order to drive the hand to the grasp pose. The vision controller uses virtual visual servoing to compute the matrix transformation between the external camera and the door handle and implements a position-based visual servoing control to guide the robot until the hand touches the door handle. The tactile controller develops a control law which aligns the fingertips with the planar surface of the door handle. The three control velocities computed by these controllers are combined by a hybrid control approach which uses each velocity for a given Cartesian direction. In particular, this hybrid controller is implemented by three mutually orthogonal matrices which determine the unique direction where each controller (position, vision or tactile) is applied. The velocity obtained from this position-vision-tactile controller is then modified by an impedance force controller which ensures that the robot hand is applying the force required to open the door. In addition, this control strategy uses the tactile information to balance the pressure between the fingertips. Thereby, the contact is kept without generating too high pressure values which may damage the sensors or the robot and which are not considered by previous vision-force approaches. Table 5 summarizes the multi-sensor control strategies developed by Spanish researchers which have been described in Section 5.

## 6. Conclusions

This paper presents a detailed review on the control strategies developed by Spanish researchers which are used to control the movements of robotic systems depending on the information registered by sensors. As vision, force and tactile sensors are the most commonly used sensors to analyze how robots interact with the environment, the corresponding control strategies have been exposed: visual servoing control, force control and tactile control. In particular, three different techniques have been presented for implementing visual control: position-based visual servoing, image-based visual servoing and stereo visual servoing. Similarly, direct and indirect force control techniques have been enumerated in order to describe force control. Tactile control techniques have been organized according to their application in robotic tasks: object identification and manipulation control. For each of these control techniques, not only their theoretical basis has been explained but also the sensor configuration employed for their implementation and their final application.

This in-depth study permit us to develop an analysis of current research on robotic sensory control and an estimation of future research lines which will be based on it. The great number of works based on visual servoing demonstrates that vision is and will be the most widespread control strategy for adapting robotic systems to their environment. New visual servoing controllers will be based not only on new algorithmic improvements to the current techniques (such as new online estimators for the interaction matrix) but also on new sensors (such as omni-directional cameras which increase the field of view) and new visual features (such as structured light patterns which are independent of the object appearance). However, visual servoing is not sufficient for developing correctly robotic tasks which require physical interaction. Therefore, more and more multi-sensor systems based on vision, force and tactile sensors will be developed in the future. These multi-sensor systems will involve parallel advances in visual servoing and force/tactile control. On the one hand, direct visual servoing will be more widespread because it is able to control the dynamics of the robot which have an important influence on the contact forces between the robot and the object. On the other hand, force and, especially, tactile information will be integrated in the robot controller so that contact properties are not neglected.

## Figures and Tables

**Table 1.** Summary of Spanish research on visual servoing.

**Ref.**	**Sensors**	**Technique**	**Application**
[9]	Eye-in-hand configuration.	Feedforward neural network.	Industrial inspection.
[10]	Eye-in-hand configuration.	Reinforcement learning-based neural network.	Grasping of an object on a table.
[11]	Eye-in-hand configuration	Reinforcement learning-based neural network.	Autonomous submarine for underwater cable inspection tasks
[12]	Eye-in-hand configuration.	Discrete time Cellular Neural Networks	Test of the proposed visual servoing scheme.
[14] [15]	Eye-in-hand and eye-to-hand configuration.	Image-based visual servoing.	Visual servoing open architecture.
[16]	Simulated camera.	- Image-based visual servoing. - Position-based visual servoing.	Visual servoing simulator in Matlab/Simulink.
[17]	Eye-in-hand configuration.	Position-based visual servoing with change of the camera-object frame.	Simple tests of the proposed approach.
[18] [43]	Eye-in-hand mono and stereo rig configuration.	- Image-based visual servoing. - Position-based visual servoing.	Test of the proposed algorithms.
[19]	Eye-in-hand stereo rig configuration.	- Stereo image-based visual servoing with 2D point and major axis orientation features. - Position-based visual servoing.	Testbed for a classic position-based scheme.
[20]	Eye-in-hand configuration.	Position-based visual servoing.	Testbed for a classic position-based scheme.
[21]	Eye-in-hand configuration.	- Position-based visual servoing. -Motion object estimator.	Test of the proposed controller.
[22]	Eye-to-hand configuration.	Position-based visual servoing.	Internet Tele-Lab for learning visual servoing techniques.
[23]	Eye-in-hand configuration.	Position-based visual servoing.	Testbed for an autonomous satellite repairer.
[24]	Eye-in-hand configuration.	Position-based direct visual servoing.	Visual control of a 2 degree of freedom robot.
[25] [26] [27]	Eye-in-hand configuration.	- Position-based direct visual servoing. - Motion object estimator.	RoboTenis: a parallel robot playing table tennis.
[28]	Eye-in-hand configuration	-Position-based visual servoing based on the scene reconstruction using homographies	Mobile robot navigation
[32] [33] [34]	Eye-to-hand stereo rig configuration.	Image-based visual servoing from online estimated interaction matrix by using the properties of the epipolar geometry.	Test of the proposed online interaction matrix estimation.
[35]	Eye-in-hand	Image based visual servoing with a complete interaction matrix without stability problems and using head movements if a stability problem is detected	Aibo visual control to track the ball in a RoboCup soccer match
[36]	Eye-in-hand configuration.	Image-based visual servoing with online camera calibration.	Test of the proposed algorithm.
[39] [40] [41]	Eye-in-hand configuration.	Image-based visual servoing solving the visibility problem.	Test of visual servoing tasks with outliers.
[42]	Eye-to-hand fisheye stereo ring configuration.	Image-based visual servoing with panoramic cameras.	Safety issues for a robot arm moving in close proximity to human beings.
[44] [45] [46]	Eye-in-hand configuration.	Image-based visual servoing with structured light external visual features.	Plane-to-plane positioning tasks.
[48]	Eye-in-hand configuration.	Image-based visual servoing based on the homography decomposition.	Simulation of the proposed control scheme.
[49]	Eye-in-hand configuration	- Switched homography-based visual control	Mobile robot navigation
[50]	Eye-in-hand configuration	- Image-based visual servoing with epipole geometry as visual features. - Switching control law to deal with the motion constraints of the platform	Mobile robot navigation
[51]	Eye-in-hand configuration	Image-based visual servoing with epipoles as visual features	Mobile robot navigation
[52]	Eye-in-hand configuration	Motion estimator from multiple planar homographies	Vision based control of unmanned aerial vehicles (UAV)
[53]	Eye-in-hand configuration	- Image-based visual servoing with epipole geometry as visual features. - Sliding mode control to resolve the singularities due to the decoupling of the matrix of the system	Mobile robot navigation
[54]	Eye-in-hand configuration	Tracking of a line with Kalman filter predictor	Autonomous submarine for underwater cable inspection tasks
[55] [56]	Eye-in-hand camera configuration.	- Image-based visual servoing. - Image path tracker based on movement flow.	Tracking of predefined paths.
[57] [58]	Eye-in-hand camera configuration.	- Image-based visual servoing. - Image path tracker based on virtual visual servoing.	Tracking of predefined paths in the change of a fault light bulb.
[61] [62]	Eye-in-hand stereo rig configuration.	- Stereo image-based visual servoing with stacked-mono and stereo-real interaction matrices. - Position-based visual servoing.	Test of the proposed algorithms.
[63]	Eye-in-hand stereo rig configuration.	- Stereo image-based visual servoing with disparity features. - Position-based visual servoing.	Test of the proposed algorithms.
[64]	Eye-in-hand stereo rig configuration.	Stereo image-based visual servoing with grasping points features.	Grasping of different objects.
[65] [66] [67]	Eye-in-hand configuration	- Image-based visual servoing. - Tracking of the features by using Lucas-Kanade approach.	Visual servoing of an autonomous helicopter
[71]	Eye-to-hand configuration.	- Image-based visual servoing. - Motion object estimator.	Automatic chaser car in a slot game.
[72]	Eye-in-hand configuration.	- Image-based visual servoing. - Motion object estimator.	Peg-in-hole task in motion.
[73]	Eye-in-hand configuration.	- Image-based visual servoing. - Motion object estimator.	Tests of the proposed motion estimator.
[75]	Eye-in-hand configuration	- Image-based visual servoing. - Motion object estimator.	Tracking of a desired path in the image.
[76]	Eye-in-hand configuration	- Image-based visual servoing. - Motion object estimator.	Tracking of a mobile object placed at a turntable.

**Table 2.** Summary of Spanish research on force control.

**Ref.**	**Sensors**	**Technique**	**Application**
[78]	Wrist six axis force/torque sensor.	Neural networks.	Fine motion assembly tasks.
[79] [80]	Wrist DSP-based six axis force/torque sensor.	Impedance control.	Test of a contact force estimator.
[81] [83]	Wrist six axis force/torque sensor.	Impedance control.	Test of a self-calibrated contact force estimator.
[84]	Wrist six axis force/torque sensor.	Hybrid force/motion control.	Bone drilling in a surgical repairing task.
[86] [89]	Built-in strain gauges.	Proportional pure force control.	Control of a climbing and walking robot.
[88]	Built-in strain gauge placed at the beginning of the link.	- Switch motion/force control. - Modified PID force control.	Control of free and constrained motion of a flexible robot.
[91]	Wrist force/torque sensor.	Geometric analytical models.	Fine motion assembly tasks.
[96]	Built-in strain gauges.	Admittance control with the force controller in the joint space.	Control of a legged-robot.
[97]	Wrist six axis force/torque sensor.	- Impedance control. - Hybrid force/motion control.	Open software architecture to test robot interaction tasks.
[98]	Wrist six axis force/torque sensor.	- Different direct force control schemes. - Impedance control.	Test architecture for the analysis of the mechanical response in car seats.
[99]	Wrist six axis force/torque sensor.	Proportional pure force control.	Screwing in an assembly task.
[103]	Wrist six axis force/torque sensor.	- Impedance control. - Hybrid force/motion control.	Robot humanoid for household furniture common tasks.
[104]	Wrist six axis force/torque sensor.	Hybrid force/motion control.	Service robot for shaving and feeding tasks.
[107]	Wrist-mounted strain gauges.	Neural networks	Fine motion assembly tasks.

**Table 3.** Classification of tactile sensors.

**Sensor Types**	**Technologies**	**Physical Properties**	**Robot Configuration**
Pressure sensing arrays	- Capacitive - Piezoresistive - Optical	Static (normal pressure)	Extrinsic
Skin deflection sensors	- Conductive rubbers - Piezoresistive strain gauges - Optical	Static (deformation)	
Dynamic tactile sensors	- Piezoelectric transducers - Accelerometers - Piezoelectric stress sensors	Dynamic (vibration, stress)	
Fingertip force/torque sensors	- Piezoresistive strain gauges	Static (force/torque)	Intrinsic

**Table 4.** Summary of Spanish research on tactile control.

**Ref.**	**Sensors**	**Technique**	**Application**
[115]	Built-in force/torque sensor based on strain gauges.	Intrinsic tactile sensing for normal vector computation.	Pipe crawling robot.
[116]	Fingertip 16×16 conductive rubber contact layer.	Method with 3 phases: noise cancellation, image processing and classification by a LVQ network.	Classification of the local shapes of objects gripped by a robotic hand.
[117] [118]	- Artificial skin on a parallel robotic gripper. - Force fingertip sensor on a robotic finger with muscles.	Neural network organized as a topographic map of joint positions and contact forces.	Grasping of objects of different stiffness with a predefined force.
[119]	- Three fingertip 8×5 pressure sensing arrays. - Wrist force/torque sensor.	Force-pressure control law for controlling the applied force and maximizing the contact surface.	Robotic assistant that picks up books in a library.
[120] [121]	- Four FSRs. - Accelerometers. - Piezoelectric sensors.	Control algorithm which detects grasping events from sensor data and generates the user's feedback.	Clinical prosthesis which provides the user with feedback.
[122]	- 16×16 Array of piezoresistive tactile sensors. - Signal conditioning circuit. - FPGA	Algorithm implemented in a FPGA which detects slipping according to the number and duration of the digital pulses obtained from the tactile sensors.	Slipping detection alarm for manipulation tasks.

**Table 5.** Summary of Spanish research on multi-sensor control.

**Ref.**	**Sensors**	**Technique**	**Application**
[58] [128] [129]	- Wrist six axis force/torque sensor. - Eye-in-hand camera configuration.	- Shared visual-force control based on the force-image interaction matrix. - Time-independent image path tracker based on virtual visual servoing.	Change of a faulty bulb in a streetlamp.
[72]	- Wrist six axis force/torque sensor. - Eye-in-hand camera configuration. - Eye-to-hand camera configuration.	- Impedance visual/force control. - General weighted shared control.	Peg-in-hole task in motion.
[123]	- Wrist six axis force/torque sensor. - Eye-in-hand camera configuration.	- Impedance visual/force control. - Image path modification based on the homography matrix.	Different interaction tasks tracking a desired path.
[124]	- Wrist six axis force/torque sensor. - Eye-in-hand camera configuration.	- Impedance visual/force control. - Surface discontinuities detection based on the homography matrix and structured light.	Different interaction tasks tracking a desired path in contact with an object.
[125]	- Wrist six axis force/torque sensor. - Eye-in-hand camera configuration.	Shared visual-force control.	Disassembly task.
[126] [127]	- Wrist six axis force/torque sensor. - Eye-in-hand camera configuration.	- Shared visual-force control. - Position-based visual servoing.	Service robot opening a door of a wardrobe.
[130]	- Wrist six axis force/torque sensor. - Eye-in-hand camera configuration.	- Shared visual-force control. - Time-independent image path tracker based on movement flow-based visual servoing.	Different interaction tasks tracking a desired path.
[131] [132]	- Wrist six axis force/torque sensor. - Eye-in-hand stereo rig camera configuration.	Visual/force hybrid control.	Library assistant robot.
[133] [134]	- Stereo-head with 5 d.o.f. - Two piezoresistive tactile skins on a robotic gripper.	Neural networks with VAM structure which relate visual and tactile data with joint positions.	Reaching and grasping tasks of unknown objects.
[135]	- Wrist six axis force/torque sensor. - 8×5 fingertip tactile array. - Eye-to-hand camera configuration.	Position-vision-tactile hybrid control modified by an impedance force control.	Service robot which opens a sliding door.
